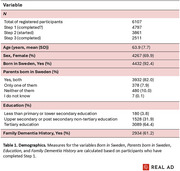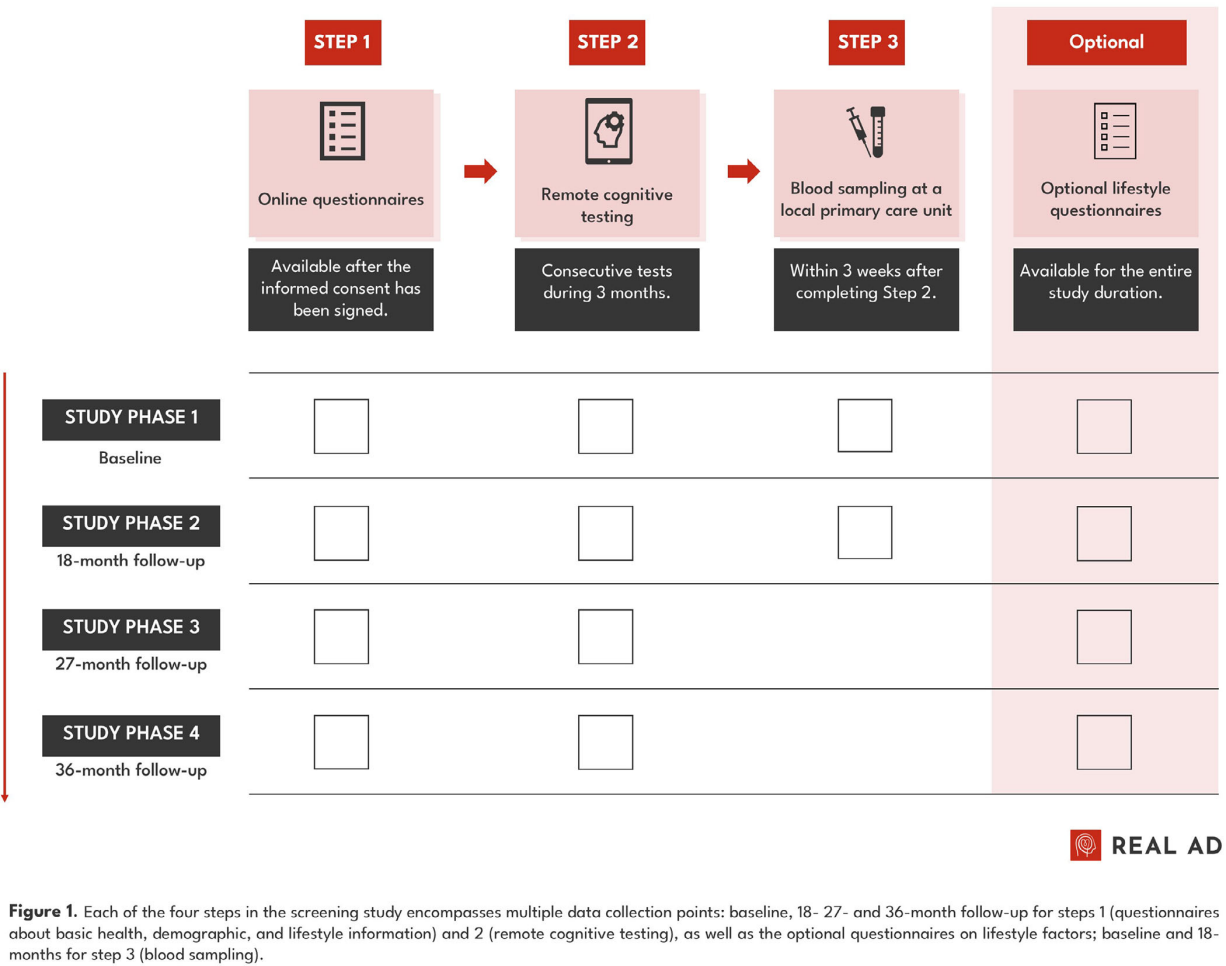# From zero to 6,000 in 9 months: Recruitment and innovation strategies for a remote longitudinal Alzheimer's population‐based screening study – Insights from the REAL AD study

**DOI:** 10.1002/alz70860_106340

**Published:** 2025-12-23

**Authors:** Iris Bosch, Frida Lenér, Laura Stankeviciute, Ellen Hanna Singleton, Fredrik Öhman, Kajsa Quitz, Maria Dottori, David Berron, Nicolai Franzmeier, Silke Kern, Henrik Zetterberg, Kaj Blennow, Michael Schöll

**Affiliations:** ^1^ Department of Psychiatry and Neurochemistry, University of Gothenburg, Mölndal, Västra Götalandsregionen, Sweden; ^2^ Region Västra Götaland, Sahlgrenska University Hospital, Department of Neuropsychiatry, Gothenburg, Västra Götalandsregionen, Sweden; ^3^ Region Västra Götaland, Research, Education, Development & Innovation (REDI), Primary Health Care, Gothenburg, Sweden; ^4^ Department of Psychiatry and Neurochemistry, Institute of Neuroscience and Physiology, Sahlgrenska Academy, University of Gothenburg, Gothenburg, Västra Götalands, Sweden; ^5^ Department of Psychiatry and Neurochemistry, University of Gothenburg, Mölndal, Västra Götalands län, Sweden; ^6^ Region Västra Götaland, Sahlgrenska University Hospital, Department of Neuropsychiatry, Gothenburg, Västragötalandsregionen, Sweden; ^7^ Region Västra Götaland, Sahlgrenska University Hospital, Department of Neuropsychiatry, Gothenburg, Västra Götalands län, Sweden; ^8^ Wallenberg Centre for Molecular and Translational Medicine, University of Gothenburg, Gothenburg, Sweden; ^9^ Närhälsan Primary Health Care, Region Västra Götaland, Gothenburg, Sweden; ^10^ Region Västra Götaland, Research, Education, Development & Innovation (REDI), Primary Health Care, Gothenburg, Västra Götalands län, Sweden; ^11^ Clinical Memory Research Unit, Department of Clinical Sciences, Lund University, Lund, Sweden; ^12^ German Center for Neurodegenerative Diseases (DZNE), Magdeburg, Germany; ^13^ Institute for Stroke and Dementia Research (ISD), University Hospital, LMU Munich, Munich, Bavaria, Germany; ^14^ Munich Cluster for Systems Neurology (SyNergy), Munich, Bavaria, Germany; ^15^ Region Västra Götaland, Sahlgrenska University Hospital, Department of Neuropsychiatry, Gothenburg, Sweden; ^16^ Department of Psychiatry and Neurochemistry, Institute of Neuroscience and Physiology, the Sahlgrenska Academy, University of Gothenburg, Molndal, Sweden; ^17^ Department of Public Health and Community Medicine, University of Gothenburg, Gothenburg, Sweden; ^18^ Clinical Neurochemistry Laboratory, Sahlgrenska University Hospital, Gothenburg, Sweden; ^19^ UK Dementia Research Institute, UCL Institute of Neurology, University College London, London, England, United Kingdom; ^20^ Department of Neurodegenerative Disease, UCL Queen Square Institute of Neurology, University College London, London, United Kingdom; ^21^ Hong Kong Center for Neurodegenerative Diseases, Hongkong, China; ^22^ University of Wisconsin School of Medicine and Public Health, Madison, WI, USA; ^23^ Clinical Neurochemistry Laboratory, Sahlgrenska University Hospital, Mölndal, Sweden; ^24^ Department of Psychiatry and Neurochemistry, University of Gothenburg, Mölndal, Sweden; ^25^ University of Gothenburg, Gothenburg, Västra Götalands län, Sweden

## Abstract

**Background:**

The REAL AD study aims to validate the diagnostic and prognostic performance of combining blood‐based biomarkers and remote cognitive testing as a screening approach for early Alzheimer's disease (AD), leveraging an existing healthcare infrastructure in Western Sweden. Here, we discuss strategies for recruiting a large and representative cohort, demographics and participant retention during the first study phase, and evaluate the process of implementing innovations enabling a fully remote study design.

**Method:**

In April 2024, a recruitment campaign was launched in collaboration with a PR company to recruit at least 3000 participants between 50‐80 years from the general population. Through an online study platform, participants were enrolled and asked to answer health and lifestyle questionnaires (Step 1), guided to remotely administered cognitive testing (Step 2; using the neotivTrials app or Cognitron battery), and blood sampling at any of the 105 public regional primary care units (Step 3; Figure 1). Additionally, optional remote blood sampling complemented the primary study protocol.

**Result:**

At abstract submission, *N* = 6092 participants (mean age=63.9 years, 70% female) were enrolled over a nine‐month period, with recruitment ongoing for another month (Table 1). Retention rates for initial study steps were *N* = 4785 participants at Step 1, *N* = 3860 at Step 2, and *N* = 2491 at Step 3. These rates align with or exceed findings from a cross‐study evaluation of retention in remote digital health studies (Pratap et al., 2020). Innovations enabling a fully remote study design were critical in achieving these recruitment and retention rates. However, attrition highlights challenges in maintaining engagement in remote study designs. Key innovations will be evaluated, focusing on: (1) an online study platform with secure authentication, (2) remote cognitive testing, (3) blood sampling via local healthcare providers, and (4) remote sample collection, all achieved through collaboration with relevant stakeholders. Structured feedback reports from participants will complement evaluations.

**Conclusion:**

REAL AD demonstrates the feasibility of implementing a large‐scale, fully remote screening approach for early AD. Preliminary results highlight the effectiveness of key innovations and stakeholder collaboration, though attrition underlines the need to address engagement barriers. Future improvements and recommendations for other remote population‐based screening initiatives will be discussed.